# Bronchoscopic removal of lower airway foreign body: a two-center retrospective cohort study

**DOI:** 10.1186/s12890-026-04258-3

**Published:** 2026-03-28

**Authors:** Shogo Toyama, Kenichiro Kudo, Kohei Fujita, Yuki Takigawa, Hiromi Watanabe, Issei Oi, Osamu Kanai, Ken Sato, Keiichi Fujiwara, Kiminobu Tanizawa, Satoru Sawai

**Affiliations:** 1 Division of Respiratory Medicine, Center for Respiratory Diseases, National Hospital Organization Kyoto Medical Center, 1-1, Fukakusa-Mukaihata, Fushimi, Kyoto-City, Kyoto, Japan; 2 Department of Respiratory Medicine, National Hospital Organization Okayama Medical Center, Okayama, Japan; 3 Department of Thoracic Surgery, National Hospital Organization Kyoto Medical Center, Kyoto, Japan

**Keywords:** Airway foreign body, Aspiration, Airway stenosis, Flexible bronchoscopy, Aging population

## Abstract

**Background:**

Tracheobronchial foreign body (FB) aspiration is a life-threatening condition, particularly in older adults. Despite its clinical significance, research on the etiology of adult FB aspiration, as well as real-world outcomes and complications associated with FB removal　in lower airways, remains limited.

**Patients and methods:**

This was a two-center retrospective cohort study in Japan. We included adult patients aged 18 years or older who underwent bronchoscopic FB removal between April 2010 and March 2025.

**Results:**

A total of 28 patients were included in this analysis (mean age, 75.4 years [median age: 75 years]; range, 55–102 years). Nineteen patients (67.9%) were male. The most frequently reported symptoms were coughing (46.4%), sputum production (39.3%), and dyspnea (17.9%). A total of 30 FBs were identified, with dental prostheses (60.0%) being the most common type, followed by natural teeth (10.0%). FBs were slightly more common on the right side (53.3%) than on the left side (43.3%), with the right lower lobe being the most frequent site of lodgement (30.0%), followed by the left lower lobe (20.7%). The most frequently bronchoscopic findings were mucosal edema (36.7%) and bleeding (26.7%). FB removal was performed using flexible bronchoscopy in 26 patients (92.9), with the exception of two cases (7.1%) in which rigid bronchoscopy was employed. Bronchoscopy was performed in the endoscopy room for 21 patients (75.0%). Among the 28 patients, standard forceps were used in 24 (85.7%), followed by basket forceps, which was employed in 10 patients (35.7%). FB removal was successful in 26 (92.9%) patients. Regarding adverse events, hypoxia was the only complication observed, occurring in 18 patients (64.3%); no other adverse events were reported.

**Conclusions:**

Most FB in the lower airways can be successfully and safely removed via flexible bronchoscopy.

**Supplementary Information:**

The online version contains supplementary material available at 10.1186/s12890-026-04258-3.

## Introduction

Tracheobronchial foreign body (FB) aspiration is a life-threatening condition most commonly observed in children and older adults [1]. The acute phase of FB aspiration often presents with a choking event, but if left undiagnosed, retained FBs may lead to chronic cough, recurrent pneumoniae, and hemoptysis [2]. Prompt extraction of aspirated FBs is crucial to alleviate acute symptoms and prevent long-term complications [3]. With an aging population in Japan, the prevalence of FB aspiration is expected to rise, particularly among older adults with impaired swallowing, cerebrovascular diseases, dementia, and those taking psychiatric medications [4]. Despite its clinical significance, research on adult FB aspiration remains limited. Thus, the aim of this study was to reveal the etiology of FB in lower airways as well as real-world outcomes and complications associated with the removal.

## Patients and methods

This was a two-center retrospective cohort study conducted at the National Hospital Organization (NHO) Kyoto Medical Center (600 beds), Kyoto, Japan, and the NHO Okayama Medical Center (600 beds), Okayama, Japan. We included adult patients aged 18 years or older who underwent FB removal performed by respiratory physicians between April 2010 and March 2025. We collected medical information from the bronchoscopy databases in the two hospitals. Participants with incomplete medical records were excluded. Background factors, chief complaints, type and location of the FB, duration of examination, type and dose of anesthesia, and complications during and after the procedure were investigated. The efficacy was evaluated based on the success rate of FB removal. Safety was assessed by monitoring the incidence of adverse events during the procedure and within 72 h post-examination. The adverse events were defined as follows: hypoxemia, a 4% decrease in oxygen saturation from baseline or the need for supplemental oxygen; hypertension, a systolic blood pressure > 160 mmHg; hypotension, a systolic blood pressure < 80 mmHg; fever, a 1℃ increase in body temperature from baseline. The severity of adverse events was assessed using the Common Terminology Criteria for Adverse Events version 5.0.

### Procedures

At our institution, all foreign bodies (FBs) were managed with an initial attempt at removal via bronchoscopy. The flexible bronchoscopes BF-1TQ290 with a 3.0 mm channel and BF-Q190 with a 2.0 mm channel (Olympus Corporation, Tokyo, Japan) were used for FB removal procedures, which were performed under local anaesthesia with midazolam. Conversely, rigid bronchoscopy (Harada Corporation, Tokyo, Japan), available only at the NHO Okayama Medical Center, was performed under general anesthesia. Additionally, a cryoprobe was available exclusively at the NHO Okayama Medical Center.

The dosage of the anesthetic drugs was determined at the discretion of the attending physician. A pulse oximeter, blood pressure monitor, and electrocardiograph were used to monitor the patient’s vital signs. The patient was placed in the supine position. Bronchoscopy was discontinued if the systolic blood pressure remained below 80 mmHg despite rapid fluid infusion; if oxygen saturation persisted below 90% despite oxygen administration at 10 L/min or more; if hypotension-inducing arrhythmias persisted; or if the attending physician determined that continuing the procedure was unfeasible.

## Results

**Fig. 1:**
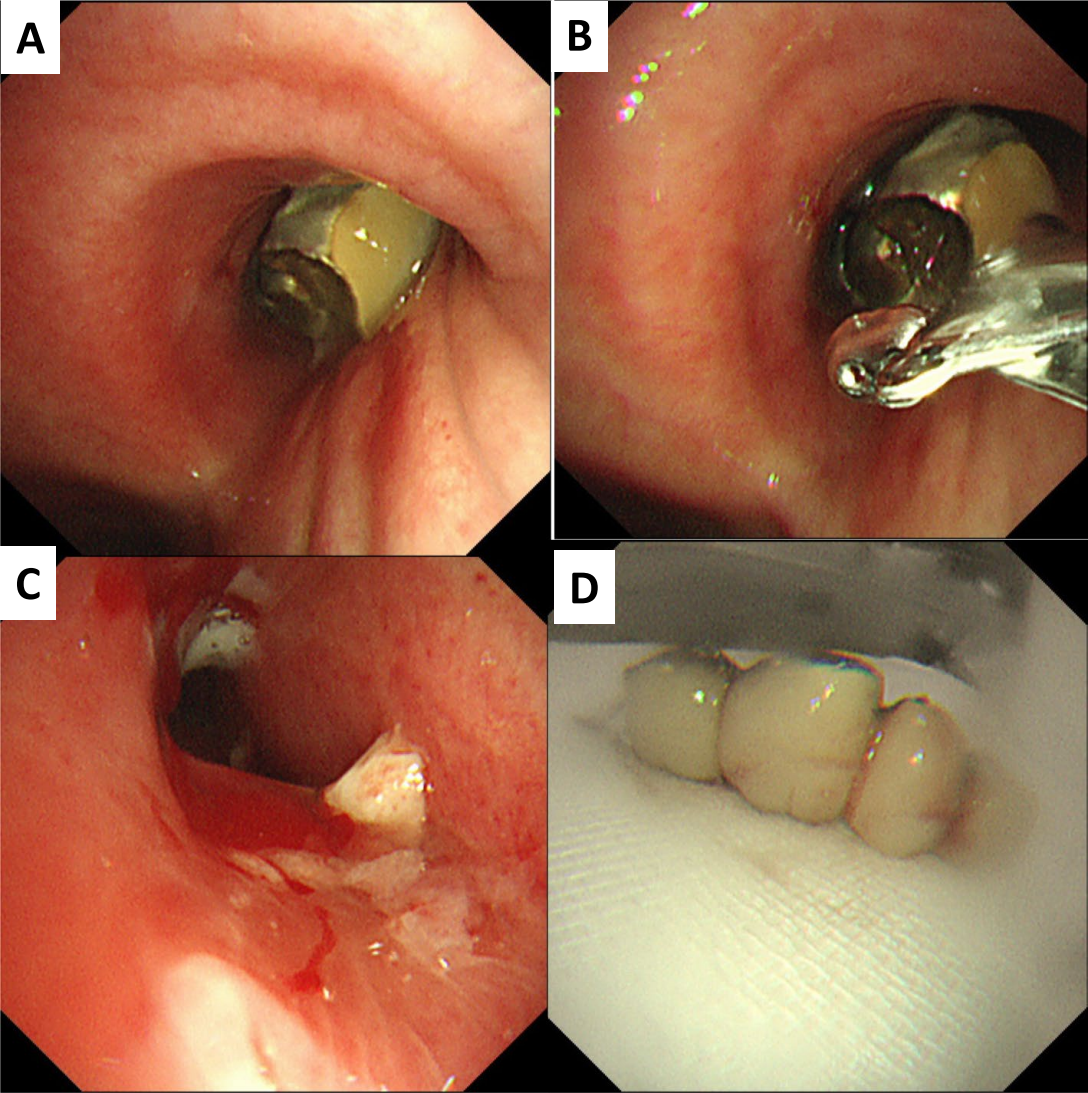
Bronchoscopic images from case KMC-12. **A** A dental prostheses was identified in the right main bronchus, accompanied by bleeding and granulation tissue formation. **B**–**D** The dental prostheses was successfully grasped and removed using standard forceps

A total of 28 patients who underwent FB removal were included in the analysis, accounting for 0.36% of all the patients who underwent bronchoscopic procedures at the two institutions. A representative example is shown in Fig. 1. The patient characteristics are summarized in Table 1. The mean age was 75.4 years (median, 75 years [range, 55–102]), with 13 patients (46.4%) aged ≥ 75 years. Nineteen patients (67.9%) were male. The mean body mass index (BMI) was 20.9. Eight patients (28.6%) had an Eastern Cooperative Oncology Group performance status of 2 or higher, and 14 patients (50.0%) had a history of smoking. The common comorbidities included hypertension (35.7%), chronic obstructive pulmonary disease (25%), diabetes mellitus (14.3%), and cerebrovascular diseases (14.3%).

**Table 1:** Characteristics of the study patients

Characteristics	*N* = 28
Age	75.0 (55.0–102.0)
Sex (Male)	19 (67.9)
Elderly (≧75)	13 (46.4)
Comorbidity	
COPD	7 (25.0)
Asthma	1 (3.6)
Interstitial pneumonia	2 (7.1)
Tuberculosis	2 (7.1)
Cardiovascular	3 (10.7)
Hypertension	10 (35.7)
Diabetes Mellitus	4 (14.3)
Cerebrovascular	4 (14.3)
Neurological Disorder	2 (7.1)
Malignancy	3 (10.7)
BMI	20.9 ± 3.8
Poor PS (≧2)	8 (28.6)
Smoking	14 (50.0)
Report of the aspiration episode	9 (32.1)
Symptoms at presentation	20 (71.4)
Sputum	11 (39.3)
Haemoptysis	1 (3.6)
Cough	13 (46.4)
Sore throat	3 (10.7)
Chest pain	1 (3.6)
Dyspnoea	5 (17.9)
Choking	0 (0.0)
Fever	5 (17.9)

Data are presented as numbers (%), medians (ranges), or mean ± SD

*Abbreviations*: *BMI* body mass index, *PS* performance status, *COPD* chronic obstructive pulmonary disease

Among the 28 patients, nine (32.1%) recalled an aspiration episode, while 20 (71.4%) were symptomatic at presentation. The most frequently reported symptoms were cough (46.4%), sputum production (39.3%), dyspnea (17.9%), and fever (17.9%). In contrast, asymptomatic patients were primarily diagnosed incidentally through chest X ray or CT imaging.

The types, locations, and the presenting findings associated with FBs are summarized in Table 2. A total of 30 FBs were identified, as multiple FBs were found in two cases. The most common type was dental prostheses (60.0%), followed by natural teeth (10.0%). Organic FBs accounted for 13.3% of all the cases. Two FBs (6.7%) were identified as sputum plugs, which were included because of the initial clinical suspicion of exogenous FBs and later clarified via bronchoscopy.

**Table 2:** Types, sites, and the presenting findings of foreign bodies (FB)

The types, places, and the presenting findings of FB	*N* = 30
Type of FB (inorganic)	
Dental prostheses	18 (60.0)
Natural teeth	3 (10.0)
Endobronchial Watanabe Spigot	2 (6.7)
Pushpins	1 (3.3)
Pills	1 (3.3)
Calcified lesions	1 (3.3)
Type of FB (organic)	
Sputum	2 (6.7)
A green soybean	1 (3.3)
Tempura of lotus root	1 (3.3)
Sites	
Trachea (%)	1 (3.3)
Right main bronchus (%)	2 (6.7)
Right upper-lobe (%)	0 (0)
Right intermediate bronchus (%)	3 (10.0)
Right middle-lobe (%)	2 (6.7)
Right lower-Lobe (%)	9 (30.0)
Left main bronchus (%)	4 (13.3)
Left upper-lobe (%)	3 (10.0)
Left lower-Lobe (%)	6 (20.0)
Complications	
Mucosal oedema	11 (36.7)
Bleeding	8 (26.7)
Atelectasis	6 (20.0)
Granulation formation	5 (16.7)
Obstructive pneumonia	5 (16.7)

Data are expressed as numbers (%)

FBs were slightly more common on the right side (53.3%) than on the left side (43.3%), with the right lower lobe being the most frequent site of lodgement (30.0%), followed by the left lower lobe (20.0%). The most commonly observed findings during bronchoscopy were mucosal edema (36.7%), bleeding (26.7%), and, granulation tissue formation (16.7%). Chest computed tomography (CT) revealed atelectasis in 20.0% of FBs and obstructive pneumonia in 16.7%.

FB removal was performed using flexible bronchoscopy in 26 patients (92.9%). In the remaining two cases (7.1%), rigid bronchoscopy was employed due to the size or sharpness of the FBs. Bronchoscopy was performed in the endoscopy room for 21 patients (75.0%). The remaining procedures were performed in the operating room (three patients, 10.7%), intensive care unit (two patients, 7.1%), emergency room (one patient, 3.6%), and general ward (one patient, 3.6%), primarily due to the patients’ critical conditions.

The details of the FB removal procedure are summarized in Table 3. Among the 28 patients, standard forceps were used in 24 cases (85.7%), followed by basket forceps in 10 (35.7%) and a curette in three (10.7%). No cryoprobes were used in any case. FB removal was successful on the first attempt in 24 patients, with an overall success rate of 92.9% (26 patients). The mean dose of midazolam administered was 4.5 ± 3.5 mg, and the average procedure time was 36.4 ± 19.7 min. Hypoxia was the only complication observed, occurring in 18 patients (64.3%). No other adverse events such as hypertension, hypotension, or fever were reported. The median hospitalization duration was 6 days (range, 2–840 days).

**Table 3:** Details of the foreign body (FB) removal procedures

Details of procedure	*N* = 28
Bronchoscopy	
Flexible bronchoscopy	26 (92.9)
Rigid bronchoscopy	2 (7.1)
Devices used for foreign body removal	
Standard forceps	24 (85.7)
Basket forceps	10 (35.7)
Curette	3 (10.7)
Balloon	2 (7.1)
Alligator forceps	1 (3.6)
Brush	1 (3.6)
Cryoprobe	0 (0)
Sedation	
Midazolam	4.5 ± 3.5 mg
Procedure time	36.4 ± 19.7 min
Adverse events	
Hypoxia	18 (64.3)
Hypertension	0 (0)
Hypotension	0 (0)
Fever	0 (0)
Results	
First attempt success	24 (85.7)
Overall success	26 (92.9)
Days of hospitalization	6 (range: 2–840 days)

Data are shown as numbers (%), medians (ranges), or mean ± SD

Adverse events were defined as follows: hypoxaemia (a 4% decrease in oxygen saturation from baseline or the need for supplemental oxygen), hypertension (systolic blood pressure > 160 mmHg), hypotension (systolic blood pressure < 80 mmHg), fever (an increase in body temperature of 1℃ from baseline)

Multiple devices were sometimes used in a single procedure

For detailed data per institution and per patient, please see the Supplemental Files (Tables S1 and S2).

## Discussion

In adults, FB removal accounts for around 0.25–0.33% of all bronchoscopic procedures [5–9]. FB aspiration is slightly more common in males (65.9–71.1%) [2, 6, 10–14], which is consistent with our findings showing a male predominance of 67.9%. The median age reported in most studies ranges from 65 to 75 years, with aspiration risk increasing among the older adults [5, 12–14]. In comparison, the median age in our cohort was slightly higher at 75 years, which may reflect Japan's aging population and the associated healthcare challenges.

From the literature review, approximately 30% of patients (24.4%–31.1%) were unable to recall a history of aspiration at their initial medical visit [2, 10, 14]. In contrast, our study found that 67.8% of patients could not recall an aspiration episode. This discrepancy may be partly explained by the older age distribution in our cohort. Previous studies have reported delayed diagnoses, with only about 10% of patients diagnosed within 3 days of aspiration, while more than half were diagnosed more than one month later [11]. Although we attempted to collect data on time to diagnosis, these data were largely unavailable because many patients did not recall the aspiration event. The most commonly reported symptom was cough, occurring in approximately 58.5%–67.8% of cases, followed by sputum (17.4–57.6%), dyspnea (12.0–44.2%), fever (8.0%−27.1%), and hemoptysis (10.2–17.4%) [2, 11, 12, 14]. These trends generally align with our findings, except for a notably lower incidence of hemoptysis (3.6%) in our cohort.

Organic FBs, particularly food items, were predominant in numerous studies [2, 5, 10, 11, 13]. Metallic FBs, including pins, dental appliances, and plastic materials, also accounted for a significant proportion of FBs, although their prevalence varied across studies. In contrast, organic FBs constituted only approximately 10% of the cases in the present study. This inconsistency may be attributed to the aging demographics of our cohort, in which dental prostheses (60.0%) and natural teeth (10.0%) were the most common types of FBs. Differences in dietary habits may also be a contributing factor, as Japanese individuals are less likely to consume foods that are easily aspirated, such as bones, nutshells, and beans except for soybeans [15].

Previous studies have reported that both organic and inorganic FBs are most commonly found in the right bronchial tree, particularly in the right lower lobe, because of the more vertical orientation of the right main bronchus [2, 5, 6]. This is in accordance with our findings, in which FBs were more frequently found on the right side (53.3%), with the right lower lobe being the most common site of lodgement (30.0%). Regarding FB-related complications, previous studies have identified prolonged retention as the primary cause of granulation tissue formation, along with obstructive pneumonia, atelectasis, and bronchial stenosis [6, 8] In contrast, the most frequent findings observed in our cases were mucosal edema (36.7%) and bleeding (26.7%), which are typically associated with the early stages of FB aspiration. Thus, although patients were unable to recall an aspiration episode, airway FBs were suspected based on clinical symptoms and managed in a timely manner.

Rigid bronchoscopy ensures a safe airway and eliminates the risk of injury from the sharp edges of the FBs. However, the use of rigid bronchoscopy is limited to specialized facilities. Although methods for FB removal varied largely across studies, advances in technology have led to flexible bronchoscopy gradually replacing rigid bronchoscopy as the most used technique in adults [3]. A systematic review showed that in adults, flexible bronchoscopy obviates the need for rigid bronchoscopy in nearly 90% of cases [6]. In our study, FB removal was performed using flexible bronchoscopy in almost all patients, with the exception of two cases (6.7%).

In a previous study, shark-tooth forceps (44.9%) were the most common forceps used to extract FB, followed by alligator forceps (32.6%) [6]. In our study, standard forceps were primarily used. Recently, a wide variety of instruments has become available, including basket forceps, which are particularly useful for removing fragile or smooth FBs. We employed basket forceps in 10 patients (35.7%). Moreover, cryotherapy has been widely used [16], as it can effectively remove FBs with sufficient water content for freezing, even when they are encapsulated by granulation tissue due to prolonged retention or are too large to be grasped with forceps [17–19]. While cryotherapy is available at the NHO Okayama Medical Center, it has not yet been used there for FB removal.

The first attempt at FB removal was successful in nearly 90% of cases in previous studies, with almost all remaining cases successfully resolved in subsequent attempts [6, 12, 14]. Similarly, our study demonstrated a high overall success rate of 92.9%. A systematic review identified bleeding as the most common complication during flexible bronchoscopy, followed by slippage of the FB into the gastrointestinal tract, migration into another bronchial segment, and hypoxia [6]. In contrast, hypoxia (64.3%) was the only complication observed in our study, with no other adverse events, including bleeding, reported. In our study, hypoxemia was defined as either a ≥ 4% decrease in oxygen saturation from baseline or the requirement for supplemental oxygen and in most cases, the decrease in SpO₂ was mild (approximately 5%) and transient. This procedure safety may be partly attributable to the earlier stage at which the patients in our cohort presented to the hospital.

The same review reported that 89.2% of patients were managed in an outpatient setting and discharged on the same day [6]. In contrast, the median duration of hospitalization in our study was 6 days. This difference may reflect the advanced age of our patients, among whom the disuse syndrome may have contributed to the prolonged hospital stay. The patient with a hospital stay of 840 days was bedridden and required prolonged hospitalization due to underlying conditions unrelated to the foreign body removal.

This study has several limitations. First, it was a retrospective analysis conducted at two centers, which may have introduced a selection bias. Outcomes were likely influenced by the availability of specific devices at each facility, highlighting an inherent limitation in comparing procedural choices and outcomes. This limitation underscores the need for large-scale retrospective or prospective studies to validate and expand our findings. Furthermore, owing to the retrospective design, the selection of devices was left to the discretion of the attending physicians, potentially resulting in variability in device usage under similar clinical conditions.

In conclusion, we conducted a two-center retrospective cohort study on airway FB removal in adult patients. Most FB in the lower airways can be successfully and safely removed via flexible bronchoscopy. We believe that our findings contribute to a better understanding of the etiology and clinical management of airway FBs and may support future research aimed at improving diagnostic and therapeutic techniques.

## Supplementary Information

Supplementary Material 1.

## Data Availability

The data that support the findings of this study are available from the NHO Kyoto Medical Center Ethical Committee, but restrictions apply to the availability of these data, which were used under license for the current study, and so are not publicly available. Data are however available from the authors upon reasonable request and with permission of the NHO Kyoto Medical Center Ethical Committee.

## Abbreviations

FB: Foreign body
CT: Computed tomography
NHO: National Hospital Organization
