# Supplementary material for: Bronchoscopic removal of lower airway foreign body: a two-center retrospective cohort study
Source: BMC Pulm Med. 2026 Mar 28;26:213. doi: 10.1186/s12890-026-04258-3 (PMC13151087; doi:10.1186/s12890-026-04258-3)
Supplement: Supplementary file 1 — Supplementary Material 1. [file 12890_2026_4258_MOESM1_ESM.docx]

**Supplemental Files**

**Table S1. Detailed information regarding the types, sites, and complications of foreign bodies (FB)**

|  | Age,  Sex | Types of FB | Sites | Complications |
| --- | --- | --- | --- | --- |
| KMC-1 | 80, M | Dental prostheses | Left lower-lobe bronchus |  |
| KMC-2 | 85, F | 2 natural teeth | Right B9 and Right B10 |  |
| KMC-3 | 76, M | Dental prostheses | Right lower-lobe bronchus |  |
| KMC-4 | 55, M | 2 EWSs^a)^ | Left B3 and Left B3 | Granulation formation |
| KMC-5 | 82, M | Dental prostheses | Right lower-lobe bronchus | Obstructive pneumonia, Mucosal edema, Bleeding |
| KMC-6 | 68, F | A calcified lesion | Left B3 | Obstructive pneumonia, Mucosal edema |
| KMC-7 | 74, M | Green soybeans | Right intermediate bronchus |  |
| KMC-8 | 86, M | Pills | Right lower-lobe bronchus | Obstructive pneumonia, Mucosal edema |
| KMC-9 | 72, F | Dental prostheses | Bifurcation of right B8 and B9 |  |
| KMC-10 | 92, F | Dental prostheses | Right middle-lobe bronchus | Obstructive pneumonia, Atelectasis, Mucosal edema, Bleeding, Granulation formation |
| KMC-11 | 80, M | Dental prostheses | Right main bronchus | Obstructive pneumonia, Atelectasis, Mucosal edema, Bleeding |
| KMC-12 | 102, F | Dental prostheses | Right main bronchus | Bleeding, Granulation formation |
| KMC-13 | 56, M | Dental prostheses | Left main bronchus | Mucosal edema, Bleeding, Granulation formation |
| KMC-14 | 86, F | Sputum^b)^ | Trachea | Atelectasis |
| OMC-1 | 91, F | Dental prostheses | Right intermediate bronchus | Mucosal edema |
| OMC-2 | 70, M | Sputum^b)^ | Right lower-lobe bronchus | Atelectasis |
| OMC-3 | 61, F | Natural tooth | Left main bronchus |  |
| OMC-4 | 78, M | Dental prostheses | Left lower-lobe bronchus | Atelectasis, Mucosal edema, Bleeding |
| OMC-5 | 69, M | Pushpins | Right basal bronchus | Mucosal edema, Bleeding |
| OMC-6 | 83, M | Dental prostheses | Right intermediate bronchus |  |
| OMC-7 | 65, M | Dental prostheses | Left main bronchus |  |
| OMC-8 | 61, F | Lotus root tempura | Left main bronchus | Obstructive pneumonia, Atelectasis, Mucosal edema, Bleeding |
| OMC-9 | 69, M | Dental prostheses | Left basal bronchus |  |
| OMC-10 | 85, M | Dental prostheses | Left basal bronchus | Mucosal edema |
| OMC-11 | 72, M | Dental prostheses | Left lower-Lobe bronchus |  |
| OMC-12 | 62, M | Dental prostheses | Bifurcation of right B9 and B10 |  |
| OMC-13 | 82, M | Dental prostheses | Left lower-Lobe bronchus | Mucosal edema |
| OMC-14 | 68, M | Dental prostheses | Bifurcation of right B9 and B10 |  |

KMC and OMC refer to Kyoto Medical Center and Okayama Medical Center, respectively, and were used to designate the case numbers for the patients included in the study.

Abbreviations: M, male; F, female; EWS = Endobronchial Watanabe Spigot.

^a)^Although two EWSs were inserted, they were no longer needed as the pneumothorax was completely cured. Therefore, we attempted to remove these "foreign bodies".

^b)^Although an extrinsic FB was initially suspected based on CT findings, sputum was identified during bronchoscopy, and suctioning alone was performed.

**Table S2. Detailed information of the foreign body (FB) removal procedures in the two centers.**

|  | Device | MDZ (mg) | Procedure Time (min) | AE | Results | Hospitalization days |
| --- | --- | --- | --- | --- | --- | --- |
| KMC-1 | Standard forceps | N/A | N/A | - | Succeeded | 7 |
| KMC-2 | Standard forceps | N/A | 20 | Hypoxia | Succeeded | 840 |
| KMC-3^b)^ | Standard forceps | N/A | N/A | - | Succeeded | 413 |
| KMC-4^a)^ | Standard forceps | 20 | N/A | Hypoxia | Succeeded | 55 |
| KMC-5^a)^ | Standard forceps, Balloon, Basket forceps | 6 | 60 | - | 1^st^: failed; 2^nd^: succeeded | 8 |
| KMC-6 | Standard forceps, Curette, Brush | 3 | 14 | - | Failed | 2 |
| KMC-7 | Standard forceps | 4 | 20 | Hypoxia | Succeeded | 2 |
| KMC-8 | Standard forceps, Basket forceps | 2.5 | 50 | - | 1^st^: failed, 2^nd^: succeeded | 58 |
| KMC-9 | Standard forceps, Basket forceps | 4 | 40 | Hypoxia | Succeeded | 0 |
| KMC-10^a)^ | Standard forceps, Basket forceps | 6 | 49 | Hypoxia | Succeeded | 15 |
| KMC-11 | Standard forceps | 3 | 18 | - | Succeeded | 21 |
| KMC-12 | Standard forceps, Basket forceps | 0 | 16 | Hypoxia | Succeeded | 4 |
| KMC-13^b)^ | Standard forceps, Balloon, Basket forceps, Alligator forceps | 0 | 70 | Hypoxia | Succeeded | 3 |
| KMC-14 | - | 3 | 12 | Hypoxia | Succeeded | 67 |
| OMC-1 | Standard forceps | 3 | 23 | Hypoxia | Failed | 2 |
| OMC-2 | - | 5 | 16 | Hypoxia | Succeeded | 3 |
| OMC-3 | Standard forceps, Balloon | 3 | 45 | Hypoxia | Succeeded | 6 |
| OMC-4 | Standard forceps, Basket forceps | 6 | 87 | Hypoxia | Succeeded | 15 |
| OMC-5^c)^ | Grasping forceps for rigid scope | 5 | 38 | - | Succeeded | 7 |
| OMC-6 | Standard forceps | 3 | 23 | Hypoxia | Succeeded | 2 |
| OMC-7 | Standard forceps, Basket forceps | 5 | 25 | Hypoxia | Succeeded | 3 |
| OMC-8 | Standard forceps, Basket forceps | 4 | 37 | Hypoxia | Succeeded | 10 |
| OMC-9 | Standard forceps | 3 | 45 | - | Succeeded | 2 |
| OMC-10 | Standard forceps, Basket forceps, Curette | 5 | 36 | - | Succeeded | 2 |
| OMC-11 | Standard forceps, Curette | 6 | 56 | Hypoxia | Succeeded | 8 |
| OMC-12 | Standard forceps | 3 | 23 | Hypoxia | Succeeded | 2 |
| OMC-13 | Standard forceps | 5 | 21 | Hypoxia | Succeeded | 3 |
| OMC-14 | Grasping forceps for rigid scope | 5 | 65 |  | Succeeded | 8 |

MDZ = Midazolam, N/A = Not Available

^a)^ Patients with KMC-4, KMC-5, KMC-10, and OMC-4 were intubated for the duration of the bronchoscopy.

^b)^In case KMC-3, the patient aspirated a dental prostheses while already receiving mechanical ventilation. In contrast, the patient in case KMC-13 required intubation and the initiation of mechanical ventilation after aspiration of the dental prostheses.

^c)^In case OMC-5 and OMC-14, the foreign body was removed using rigid bronchoscopy while the patient was on mechanical ventilation.
